# Case report: rapid progression of conduction system disturbance in unicuspid aortic valve stenosis

**DOI:** 10.1093/ehjcr/ytaf394

**Published:** 2025-08-08

**Authors:** Aimee McCoubrey, Niki Walker, John Dreisbach, Mark Danton

**Affiliations:** Scottish Adult Congenital Cardiac Service, Golden Jubilee National Hospital, Agamemnon Street, Clydebank G81 4DY, Scotland, UK; Scottish Adult Congenital Cardiac Service, Golden Jubilee National Hospital, Agamemnon Street, Clydebank G81 4DY, Scotland, UK; Scottish Adult Congenital Cardiac Service, Golden Jubilee National Hospital, Agamemnon Street, Clydebank G81 4DY, Scotland, UK; Scottish Adult Congenital Cardiac Service, Golden Jubilee National Hospital, Agamemnon Street, Clydebank G81 4DY, Scotland, UK

## Case description

A 40-year-old patient with a severely stenosed unicuspid aortic valve awaiting elective surgery presented with palpitations and exertional dyspnoea. Electrocardiogram (ECG) revealed third degree atrioventricular block (AVB) with a rate of 44bpm. ECG 6 weeks prior showed first degree AVB with a PR-interval of 276 ms, increased from 224 ms 6 months prior.

Echocardiography showed a non-dilated left ventricle (LV) with mild-moderate concentric LV hypertrophy and ejection fraction 53%. The aortic valve was unicuspid with calcification of the free margin and severe stenosis (peak gradient 87 mmHg; mean gradient 57 mmHg).

**Figure 1:**
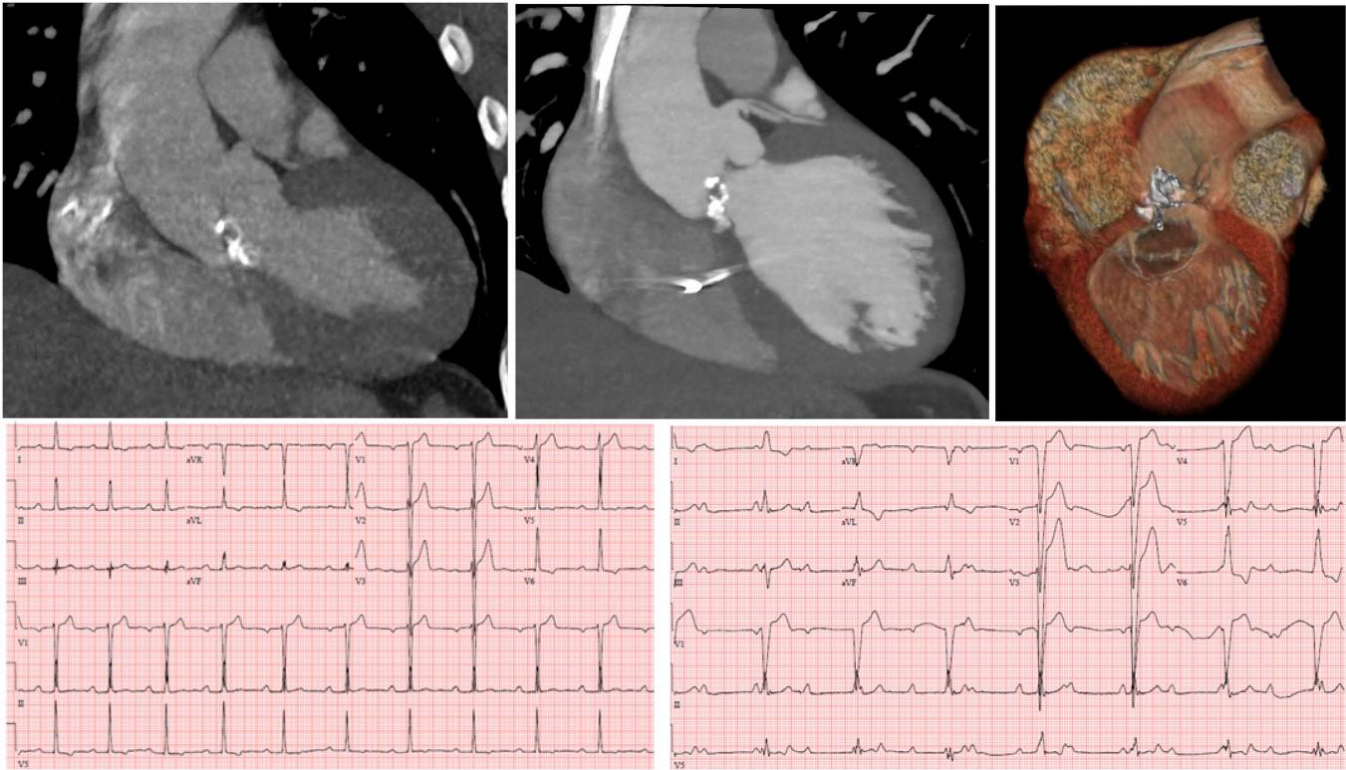
Non-gated cardiac computed tomography (CT) imaging from 6 months prior to presentation demonstrates eccentric nodular calcification of the aortic valve (*A*). Gated cardiac CT from time of presentation with complete heart block demonstrates significant extension of caseous necrotic calcification from the non-coronary cusp of the aortic valve into the LV basal anteroseptal myocardium (and pacing wire in the right ventricle) *(B, C*). Electrocardiographs showing progression of conduction disturbance from 1st degree atrioventricular block (*D*) to 3rd degree atrioventricular block (*E*).

Cardiac computed tomography (CT) showed a unicuspid aortic valve (Sievers 2), with eccentric heavy calcification extending from the non-coronary cusp into the LV basal anteroseptal myocardium in the region of the atrioventricular node—the likely culprit for development of third degree AVB (*Figure 1*).

During implantation of a temporary pacing system, he developed ventricular standstill. The patient underwent urgent inpatient surgery with a Ross procedure, ascending aorta replacement and implantation of a permanent epicardial dual-chamber pacemaker. Calcification with caseous necrosis extending into the membranous septum was observed intra-operatively.

The patient recovered well post-operatively, though remains pacing-dependent at 3 months.

It is known that aortic stenosis has potential for acute deterioration with conduction disease despite close surveillance. From our case, we have 2 learning points that will instruct the timing of surgery:

1. PR prolongation should be viewed as a potential early warning sign of rapid progression of valvular calcification.
2. In unicuspid aortic stenosis, despite a low calcific burden of the valve on echo, gated CT imaging should be performed to assess the risk to the conduction system.

## Data Availability

No new data were generated or analysed in support of this research.

## Notes

**Consent:** The authors confirm that written consent for the submission and publication of this case, including images, has been obtained from the patient in line with guidance.

## Notes

**Funding:** None declared.

